# High-Fat Diet-Induced Alterations in the Feeding Suppression of Low-Dose Nisoxetine, a Selective Norepinephrine Reuptake Inhibitor

**DOI:** 10.1155/2013/457047

**Published:** 2013-01-30

**Authors:** Nicholas T. Bello, Amy L. Walters, Jessica L. Verpeut, Priscila P. Cunha

**Affiliations:** ^1^Department of Animal Sciences, School of Environmental and Biological Sciences, Rutgers, The State University of New Jersey, 84 Lipman Drive, New Brunswick, NJ 08901, USA; ^2^Graduate Program in Endocrinology and Animal Biosciences, Rutgers, The State University of New Jersey, 84 Lipman Drive, New Brunswick, NJ 08901, USA

## Abstract

Central noradrenergic pathways are involved in feeding and cardiovascular control, physiological processes altered by obesity. The present studies determined how high-fat feeding and body weight gain alter the sensitivity to the feeding suppression and neural activation to a selective norepinephrine reuptake inhibitor, nisoxetine. Acute administration of nisoxetine (saline: 0, 3, 10, and 30 mg/kg; IP) resulted in a dose-dependent reduction in the 24 h refeeding response in male Sprague Dawley rats maintained on standard chow. In a similar fashion, nisoxetine resulted in reductions in blood pressure and a compensatory increase in heart rate. From these studies, the 3 mg/kg dose was subthreshold. In a separate experiment, however, 10 wk exposure to a high-fat diet (60% fat) resulted in weight gain and significant feeding suppression following administration of nisoxetine (3 mg/kg) compared with animals fed a control diet (10% fat). Nisoxetine (3 mg/kg) also resulted in greater neural activation, as measured by c-Fos immunohistochemistry, in the arcuate nucleus of the hypothalamus in animals exposed to the high-fat diet. Such data indicate acute nisoxetine doses that suppress food intake can impact cardiovascular measures. It also suggests that the feeding suppression to a low-dose nisoxetine is enhanced as a result of high-fat diet and weight gain.

## 1. Introduction

Obesity is a risk factor for cardiovascular disease [1, 2]. Central noradrenergic pathways are involved in regulating cardiovascular function, as well as in the control of food intake [3, 4]. Norepinephrine (NE) levels and sympathetic activity are responsive to stressors and glycemic fluctuations [5–7]. Central-acting pharmacological compounds that have actions on norepinephrine and other biogenic amines are currently used (e.g., phentermine and related formulations) or have the potential (e.g., tesofensine and bupropion/naltrexone) to treat obesity [8, 9]. However, elevations in heart rate and blood pressure are adverse effects that are often associated with the long-term use of these medications [10, 11]. Therefore, understanding how central-acting noradrenergic compounds impact feeding behavior and related neural structures can provide more targeted approaches for the treatment of obesity.

Nisoxetine (3-[2-methoxyphenoxy]-N-methyl-3-phenyl-1-propanamine) is a highly selective central-acting norepinephrine reuptake inhibitor originally developed as an antidepressant [12, 13]. In vitro studies with nisoxetine revealed that norepinephrine levels were 1,000-fold higher than serotonin (5-hydroxytryptamine; 5HT) levels and 300-fold higher than dopamine levels [13, 14]. After studies suggested it did not have the desired antidepressant effect [15], nisoxetine has been used instead as a pharmacological tool to discriminate the involvement of the noradrenergic system. Radiolabeled nisoxetine is also used to identify norepinephrine transporter (NET) kinetics and density [14, 16, 17].

Relevant to feeding behavior, nisoxetine has been used to distinguish the contribution of NET inhibition on the feeding suppression of sibutramine, a potent NE and 5HT reuptake inhibitor, and bupropion, a potent NE and dopamine reuptake inhibitor [18, 19]. Even though NE reuptake inhibition has an additive effect when combined with other monoamine reuptake inhibition, examinations of the feeding effect of nisoxetine alone have had mixed findings [18–21]. Because sibutramine and its metabolites have relatively good bioavailability when orally administered [22, 23], an apparent discrepancy between studies is the route of administration of nisoxetine. That is, in experiments where the drug is compared with sibutramine and given by oral gavage, nisoxetine has little effect on food intake [19, 21]. In contrast, nisoxetine administered by intraperitoneal (IP) injection produces a dose-dependent feeding suppression (0.1–63 mg/kg) [18]. This suggests that nisoxetine has poor oral bioavailability and has limited effectiveness on food intake when orally administered. Since the susceptibility to diet-induced obesity has been associated with increased sympathetic and central norepinephrine activity [24–26], the feeding suppression produced by an IP injection of nisoxetine can be used to determine how dietary conditions alter the noradrenergic controls of feeding. One aspect that has not been investigated is whether doses of nisoxetine that suppress food intake alter cardiovascular and blood pressure parameters.

The present studies used acute nisoxetine (IP) to identify its dose-dependent effects on feeding behavior and hemodynamic measures. From these experiments, a subthreshold dose of nisoxetine was used to determine the influence of weight gain following prolonged exposure to a high-fat diet on the noradrenergic controls of feeding. To further assess whether exposure to a high-fat diet alters neural responses to nisoxetine, c-Fos immunoreactivity was measured in several neural structures.

## 2. Materials and Methods

### 2.1. Animals

Adult male Sprague Dawley rats (8 week old) acquired from Harlan Laboratories (Frederick, MD) were individually housed and placed on a 12/12 h light dark schedule (lights off at 1700 h). Rats were fed standard chow (Purina Rat Diet 5012, 13% fat, 27% protein, 3.1 Kcal/g), unless otherwise noted, and water was available at all times during the experiments. All procedures were approved by the Institutional Animal Care and Use Committee of Rutgers University and were in accordance with NIH guidelines.

### 2.2. Nisoxetine Refeeding Dose Response

Animals (*n* = 6) were food deprived 24 h before each injection. For this within-subject design, each animal received an IP injection of vehicle (saline) and nisoxetine HCl (Tocris Biosciences; 3, 10, and 30 mg/kg). Each injection was randomized and separated by a one-week wash-out period. Food intake measurements were recorded at 0.5, 1, 4, and 24 h after injection. Food intake was measured to the nearest ± 0.1 g.

### 2.3. Nisoxetine Hemodynamic Dose Response

In an identical within-subject design as described above, animals (*n* = 12) were 24 h food deprived and, upon re-feeding and injection, cardiovascular parameters were measured by using a noninvasive tail-cuff volume pressure recording system (CODA, Kent Scientific). Animals were habituated to the CODA system for three consecutive days one week before beginning the nisoxetine or saline dosing. On recording days, animals were acclimated to the CODA system 5 min before the recordings. Measurements for each time were determined by averaging the values for 3 successful recording trials at 1, 4, and 24 h after injection. These included measurements for mean blood pressure (BP), diastolic BP, systolic BP, and heart rate.

### 2.4. Diet-Induced Changes to the Feeding Suppression of Nisoxetine

After 1-week acclimatization to standard chow, animals were placed on a high-fat diet (HF), (*n* = 9; Research Diets D12492, 60% fat, 20% protein, 5.24 Kcal/g) or control diet (CD), (*n* = 8; Research Diets D12450B, 10% fat, 20% protein, 3.85 Kcal/g) for >10 wks. Body weights were measured twice a week. After a statistical separation of body weight between groups at 10 weeks, animals began testing with a subthreshold feeding dose of nisoxetine (3 mg/kg), as determined from Section  2.2. Prior to each injection, animals were food deprived for 24 h. Half of the animals in each group were injected with nisoxetine (3 mg/kg, IP), while the other half were injected with saline (IP). In order to control for the palatability and caloric differences in diets, all animals were returned to their cages with standard chow and food intake measurements were recorded at 0.5, 1, 4, and 24 h. Immediately after the 24 h time point, all rats were returned to their respective diets (i.e., high fat or control). One week later, the animals received the counterbalance of either saline or nisoxetine.

### 2.5. Neural Activation of Nisoxetine in Animals with Diet Exposures

Two weeks following the nisoxetine feeding suppression test, animals in both diet conditions were given an IP injection nisoxetine (3 mg/kg) or saline. Groups consisted of high fat (nisoxetine); *n* = 5, high fat (Saline); *n* = 4, control diet (nisoxetine); *n* = 4, control diet (Saline); *n* = 4. Two hours later, rats were deeply anesthetized with sodium pentobarbital (100 mg/kg), exsanguinated with 0.9% saline, and perfused with 4% paraformaldehyde. Brains were removed, stored overnight in 4% paraformaldehyde with 25% (wt/vol) sucrose at 2–8°C, and then frozen and sectioned at 40 *μ*m on a cryostat through the forebrain and hindbrain regions. Sections were stored in a cryoprotectant solution at −20°C until processing. The immunohistochemistry procedure for free-floating sections with c-Fos primary antibody (1 : 20,000; Calbiochem, EMD Millipore, rabbit polyclonal, PC38) was similar to a previously published protocol [27]. To control for staining variability, each immunohistochemistry run contained matched sections from all between treatment groups and saline controls. Cell counts were performed using NIH ImageJ software with value per animal for each region representing average counts from 4 anatomically matched sections [28].

### 2.6. Statistical Analysis

For the nisoxetine dose response, food intake (Kcal) and individual hemodynamic parameters were analyzed using repeated measures ANOVA. A two-way ANOVA with repeated measures was used to determine whether exposure to the diet influenced the feeding suppression of nisoxetine. Cell counts for each region were analyzed using a factorial ANOVA to determine whether there was an interaction between nisoxetine and diet exposure. Posthoc comparisons were made when appropriate with Neuman-Keuls test, unless otherwise noted. All statistical analyses were performed with Statistica 7.1 software (StatSoft Inc.) and significance was set at *α* = 0.05.

## 3. Results

### 3.1. Effects of Acute Nisoxetine on Food Intake in 24 h Food-Deprived Chow-Fed Rats

**Figure 1:**
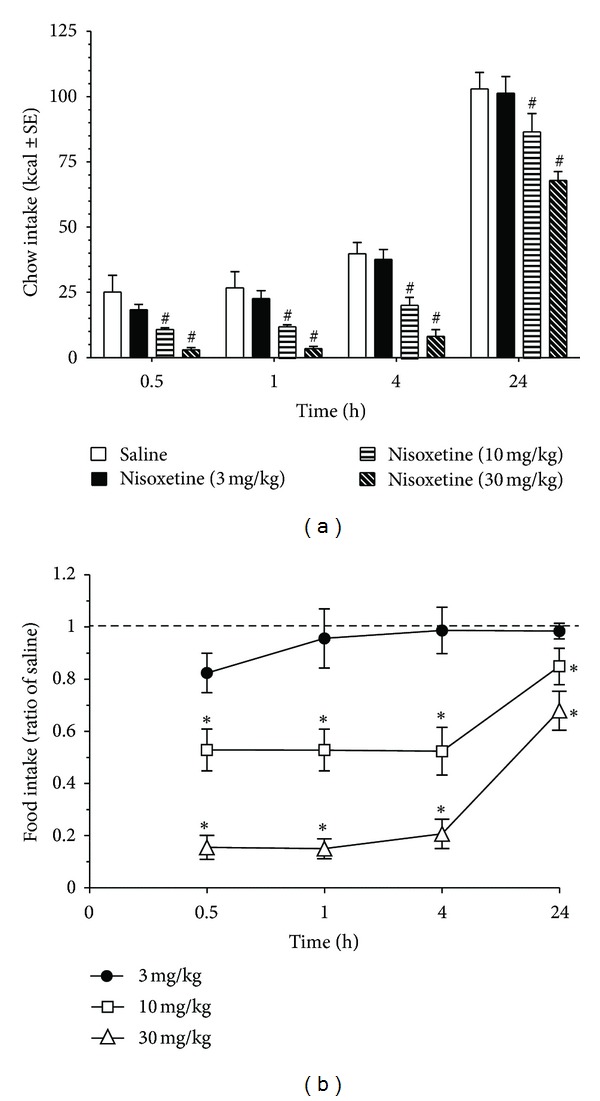
Dose response of acute nisoxetine or saline on the re-feeding intake of standard chow. Animals underwent a 24 h food deprivation prior to injections and refeeding. All animals were fed and maintained on standard chow (13% fat, 27% protein). Doses were randomly administered with a week washout between doses. (a) Intake (Kcal) shown is cumulative. # indicates differences (*P* < 0.05) from all other doses. (b) Data expressed as ratio of food intake following saline at the individual time points. Dotted line represents intake from saline injected animals (ratio of 1.0) to illustrate the feeding suppression of nisoxetine. Asterisks indicate significant differences (*P* < 0.05) from 3 mg/kg dose at respective time point.

There was a dose-dependent effect of nisoxetine (0–30 mg/kg) on the re-feeding response of standard chow. A repeated measures ANOVA indicated a dose effect (*F*(3, 15) = 14.8, *P* < 0.001), time effect (*F*(3,15) = 737.3, *P* < 0.001), and a dose X time effect that approached significance (*F*(9, 45) = 1.9, *P* = 0.05). Post-hoc testing revealed the 10 mg/kg and 30 mg/kg were significantly different from all other doses (*P* < 0.05 for both), see Figure 1(a). When data were expressed as a ratio of individual saline intake to illustrate dose suppression, see Figure 1(b), there was a significant suppression with 30 mg/kg and 10 mg/kg from 3 mg/kg at all time points (*P* < 0.05). Data indicated that the 3 mg/kg dose was subthreshold for the nisoxetine-induced feeding suppression of standard chow in animals maintained on standard chow.

### 3.2. Effects of Acute Nisoxetine on Hemodynamic Measurements in Chow-Fed Rats

**Figure 2:**
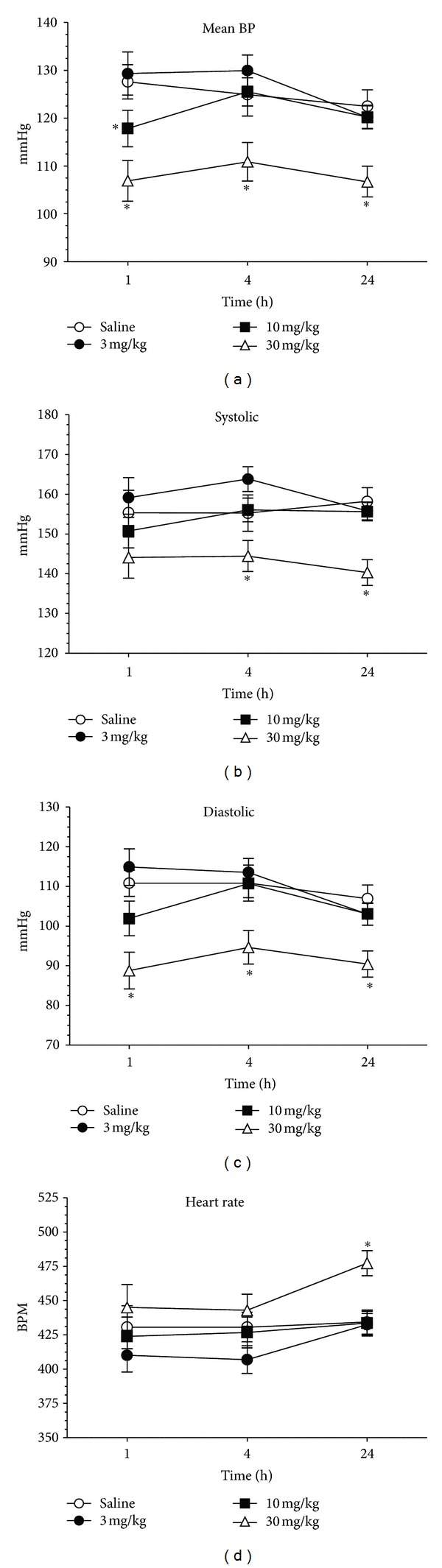
Dose response of hemodynamic measurements of acute nisoxetine or saline. Animals underwent a 24 h food deprivation prior to injections and refeeding. All animals were fed and maintained on standard chow (13% fat, 27% protein). Doses were randomly administered with a week washout between doses and tail-cuff volume pressure recording were performed at 1 h, 4 h, and 24 h. (a). Mean blood pressure (b). Systolic blood pressure (c). Diastolic blood pressure (d). Heart rate. Asterisks indicate difference from saline (*P* < 0.05) at the individual time points.

All hemodynamic measurements were significantly altered by nisoxetine. There was an overall dose effect for mean BP (*F*(3,30) = 6.1, *P* < 0.005), systolic BP (*F*(3,30) = 5.8, *P* < 0.005), diastolic BP (*F*(3,30) = 5.9, *P* < 0.005), and heart rate (*F*(3,30) = 3.2, *P* < 0.05). Post-hoc testing revealed that there was a significant decrease in mean BP at 1 h for 10 mg/kg and all time points for 30 mg/kg from saline (*P* < 0.05 for all); see Figure 2(a). The 30 mg/kg dose reduced systolic BP at 4 h and 24 h and diastolic BP at all time points from saline (*P* < 0.05 for all); see Figures 2(b) and 2(c). There was an increase in heart rate at 24 h in 30 mg/kg dose from saline (*P* < 0.05); see Figure 2(d).

### 3.3. Diet-Induced Alterations in the Feeding Suppression of Nisoxetine

**Figure 3:**
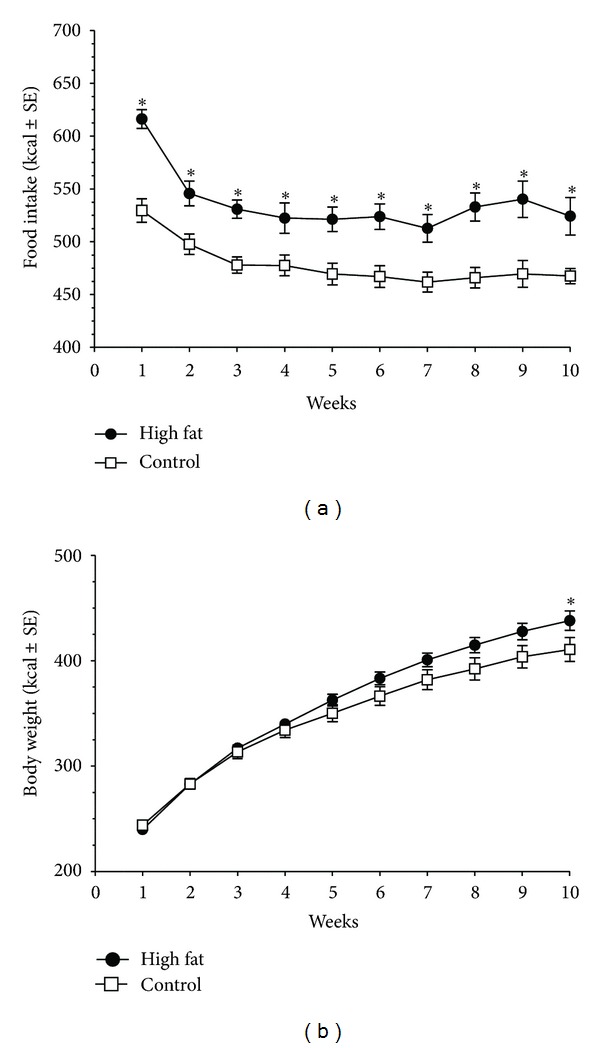
Effects of high-fat and control diets over 10 weeks. Values are means ± SE. (a) Caloric intake (Kcal) between diets, animals fed a high-fat diet (60% fat, 20% protein) had higher caloric intake than animals fed a control diet (10% fat, 20% protein) over all weeks. (b) Body weights over the 10-week feeding. Asterisks indicate group differences (*P* < 0.05) at the individual time points.

**Figure 4:**
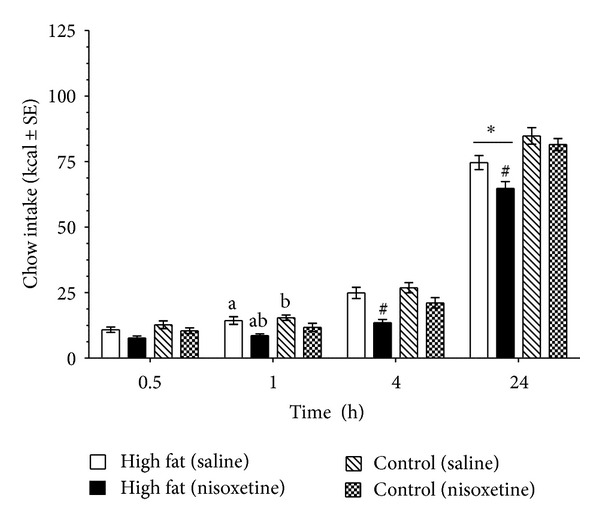
Diet-induced changes to the feeding suppression of a subthreshold nisoxetine dose. Following a 10-week feeding of high-fat or control diets, all rats were given a 24 h food deprivation and then fed standard chow. Animals were injected (IP) with either nisoxetine (3 mg/kg) or saline. One week later the injections were counterbalanced. Rats exposed to a high-fat diet showed a higher sensitivity to feeding suppression of nisoxetine at 4 and 24 hours. Animals exposed to the high-fat diet had an overall reduced intake of chow at 24 h, which is indicated by ∗ (*P* < 0.05). Similar letters indicate significance from each other (*P* < 0.05). # indicates difference (*P* < 0.05) from all groups at the individual time points.

As illustrated in Figure 3(a), there was increased caloric intake with animals on the high-fat diet. The Kcal intake showed a significant overall diet effect (*F*(1,15) = 15.4, *P* < 0.005) and time effect (*F*(9,135) = 36.2, *P* < 0.005). This was accompanied by an increase in body weight over time (*F*(9,135) = 668.4, *P* < 0.005), with a significant diet X time effect (*F*(9,135) = 6.0, *P* < 0.005). Post-hoc testing revealed that the high-fat diet group had a significantly higher body weight from the control diet group at week 10 (*P* < 0.05), see Figure 3(b). Rats with high-fat diet exposure showed a higher sensitivity to the feeding suppression of nisoxetine (3 mg/kg). There was an overall diet effect (*F*(1,15) = 13.8, *P* < 0.05) and nisoxetine effect (*F*(1,15) = 32.1, *P* < 0.005). Post-hoc testing indicated that the high-fat diet exposed group had decreased chow intake at the 24 h time point (*P* < 0.05). Nisoxetine in the high-fat diet exposed group resulted in feeding suppression from the saline at the 1 h time point (*P* < 0.05 for both). There was a greater feeding suppression from all groups with nisoxetine in the high-fat diet exposed group at the 4 and 24 h time points (*P* < 0.05 for both), see Figure 4.

### 3.4. Neural Activation with Nisoxetine in Animals with Different Diet Exposures

**Figure 5:**
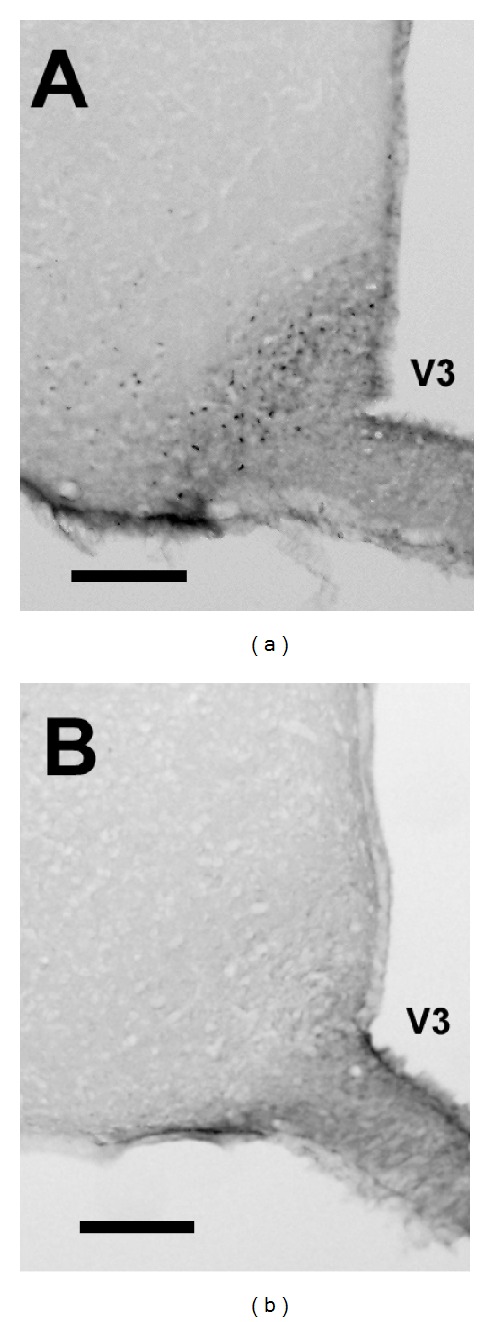
Representative coronal micrographs of immunohistochemistry c-Fos staining (black) in the arcuate nucleus of the hypothalamus. Animals exposed to a high-fat diet were injected with either nisoxetine (3 mg/kg; (a)) or saline (b) following a 24 h food deprivation. Animals were sacrificed 2 h after injection. The bar in each image represents 160 *μ*m. Coordinates for the representative sections were 3 mm caudal from bregma. Abbreviation: V3 = third ventricle.

**Figure 6:**
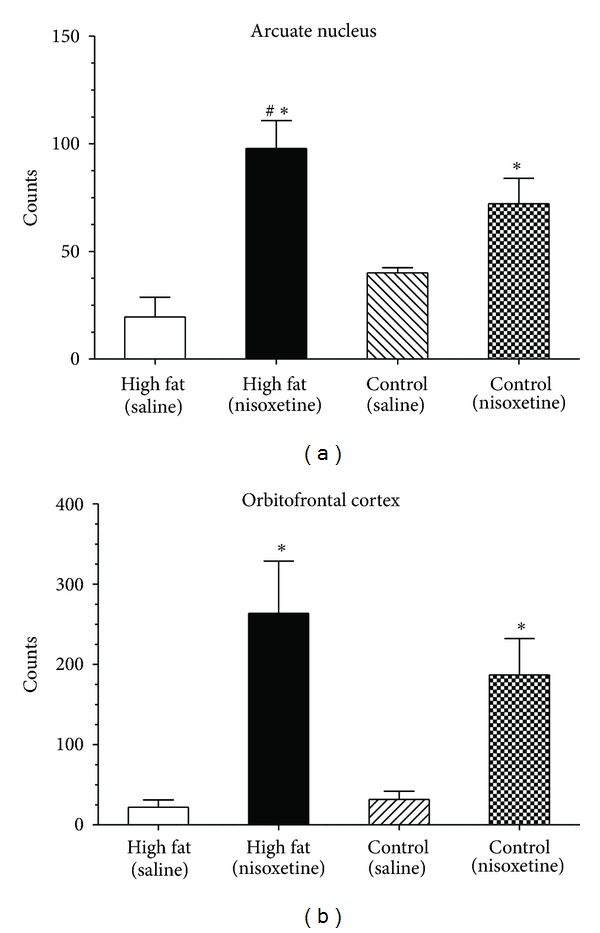
Average immunoreactive c-Fos counts in the arcuate nucleus and orbitofrontal cortex. Immunopositive cell counts means ± SE. There was an overall increase in cell number in animals injected with nisoxetine (3 mg/kg), which is indicated by ∗ (*P* < 0.05). (a) Arcuate nucleus of the hypothalamus. Planned comparisons revealed a significant effect for animals with high-fat diet exposure and nisoxetine. # indicates a difference (*P* < 0.05) between High Fat (Nisoxetine) and control diet (Nisoxetine). (b) Orbitofrontal cortex. There were no significant differences between diet conditions in this region.

Several hindbrain and forebrain regions were examined for c-Fos immunoreactivity, such as A2 cell group, locus coruleus, amygdala nuclei, and hypothalamic nuclei. The only structures to demonstrate immunopositive cells in response to nisoxetine (3 mg/kg) at the 2 h time point were the arcuate nucleus of the hypothalamus and the orbitofrontal cortex. In the arcuate nucleus, see Figures 5(a) and 5(b), there was an overall significant effect for nisoxetine (*F*(1,13) = 27.1, *P* < 0.005) and a significant diet X nisoxetine effect (*F*(1,13) = 4.8, *P* < 0.05). Post-hoc testing revealed animals injected with nisoxetine with high-fat diet exposure had more c-Fos positive cells in the arcuate nucleus than animals injected with saline and exposed to either high-fat diet or control diet (*P* < 0.05 for both). Planned comparisons between animals receiving nisoxetine revealed the high-fat diet exposed animals had significantly more c-Fos positive cells than animals exposed to the control diet (*P* < 0.05); see Figure 6(a). In the orbitofrontal cortex (lateral and ventral regions), there was only a significant effect with nisoxetine increasing the number of immunopositive cells (*F*(1,13) = 14.6, *P* < 0.05). There were no significant effects for diet or diet X nisoxetine, see Figure 6(b).

## 4. Discussion

This study investigated the acute effects on feeding and hemodynamic measures of nisoxetine, a potent selective norepinephrine reuptake inhibitor, in adult male Sprague Dawley rats. It was determined that feeding a high-fat diet, which resulted in weight gain, increased sensitivity to the feeding suppression of low-dose nisoxetine. This increased sensitivity was accompanied by enhanced neural activation in the arcuate nucleus of the hypothalamus, a neural structure critical in the homeostatic control of feeding and body weight.

The findings of this study indicated that nisoxetine, when given by intraperitoneal injection, produced a dose-dependent refeeding suppression of standard chow over 24 h. Feeding suppression from saline was observed in the 10 mg/kg and 30 mg/kg doses, while the 3 mg/kg was subthreshold in normal-weight rats fed standard chow. In a study by Billes and Cowley comparing the additive effects of nisoxetine and a selective dopamine reuptake inhibitor (GBR12783) on feeding measurements, intraperitoneal nisoxetine (0.1–63 mg/kg) alone produced a similar dose-dependent refeeding (i.e., following a 16 h food deprivation) suppression of 24 h intake of standard chow in mice [18]. In a follow-up study, Billies and Cowley found the feeding suppression of nisoxetine (4 mg/kg) did not affect locomotor activity over the 24 h feeding period, but did decrease average intrascapular temperature in mice [20]. Our investigation extended these findings over a narrower dose range in rats (3–30 mg/kg) and measured the influence of these doses on hemodynamic measurements. Our results indicated that the 30 mg/kg dose reduced mean blood pressure at 1 h, 4 h, and 24 h after injection. At the 24 h time point, the 30 mg/kg dose produced tachycardia, possibly compensatory to the depressor effect. This dose also still had an approximate 40% feeding suppression, 24 h after injection. It is interesting to note that the 10 mg/kg nisoxetine dose suppressed food intake by approximately 40% at 0.5 h, 1 h, and 4 h and only had an approximate 10% decrease of mean BP at the 1 h time point. One potential limitation to our findings is that the animals had a 24 h food deprivation prior to measuring the hemodynamic parameters. The 24 h food deprivation was done to facilitate comparisons between the feeding suppressive and hemodynamic effects of acute nisoxetine. Although prolonged food deprivation (48 h) does decrease heart rate and increase heart rate variability in lean and obese animals [29], the effects of the 24 h food deprivation in our study was controlled by randomizing the dosing scheme and having a vehicle control. Altered hemodynamic measurements and hindquarter vasodilation have been demonstrated with acute administration of sibutramine (a potent NE and 5-HT reuptake inhibitor), effects that are reported mediated by actions on the NET [30–32]. Taken together, the decrease in intrascapular temperature observed by Billes and Cowley and the predominant decrease in blood pressure in our study suggests acute nisoxetine acts centrally to produce a short-term sympathoinhibitory effect.

Another novel finding of the present study was that animals fed a high-fat diet with increased body weight, demonstrated increased sensitivity to the feeding suppression of low-dose nisoxetine. That is, feeding suppression in animals with high-fat diet exposure was observed with the 3 mg/kg dose. This nisoxetine dose did not alter the food intake in standard chow-fed animals or animals fed a low-fat control diet (10% fat). However, the cardiovascular effects of nisoxetine were not assessed in the animals exposed to the high fat diet and it is possible the sensitivity to low-dose nisoxetine on hemodynamic measures was altered as well. In previous studies, the acute feeding effects of nisoxetine (3.5 mg/kg, IP) were assessed in obese mice, but failed to demonstrate diet-induced alterations in feeding suppression [18]. One difference between studies is the method used to measure the acute feeding response. In the study by Billes and Cowley, the feeding suppressive effects of nisoxetine were measured using a high-fat diet (45% fat, 20% protein). The high-fat diet was fed as a maintenance diet for the obese mice and the lean mice were exposed to the high-fat diet about 1 week prior to testing. Because the fat content and the length of diet exposure can influence acute palatability and intake, in our study rats were maintained (>10 weeks) on either a high-fat diet (60% fat, 20% protein) or low-fat control diet (10% fat, 20% protein) and were tested on standard chow. The standard chow was not novel per se, since all animals received the diet one week prior to beginning their exposure to a high-fat or control diet. Animals with exposure to the high-fat diet demonstrated a reduced 24 h intake of standard chow, a suppressive effect that was enhanced with low dose of nisoxetine. It is possible that this alteration in the sensitivity to the feeding suppression of nisoxetine could have been masked if we had tested the animals with a more palatable high-fat diet, similar to the findings of Billes and Cowley [18]. Another possibility is that the control diet, compared with the high-fat diet, was more similar in relative macronutrient content to the standard chow (13% fat, 27% protein), so there was less of a contrast effect in the control diet-fed animals. In addition, we did not include a group fed the high-fat diet calorie matched to the control fed group, so we are unable to determine if the feeding suppression by low-dose nisoxetine was due to the high-fat diet feeding or resulting weight gain. Future studies are needed to investigate whether nisoxetine or other noradrenergic-acting agents can reduce the intake of highly palatable foods and whether weight gain can alter the noradrenergic controls of palatable foods.

The increased sensitivity in the feeding suppression of low-dose nisoxetine observed in the overweight animals suggests that the noradrenergic mechanisms involved in the neural and hormonal controls are altered in the development of diet-induced obesity. One limitation to our findings is that only a single dose of nisoxetine was used to determine the sensitivity to the feeing suppression. However, the increased sensitivity with high-fat feeding and weight gain was supported by our c-Fos immunohistochemistry studies, which demonstrated that animals exposed to the high-fat diet had greater neural activation in the arcuate nucleus of the hypothalamus. Nisoxetine also increased the number of c-Fos positive cells in the orbitofrontal cortex, but this activation was not influenced by diet. An increase in the lateral orbitofrontal cortex has been demonstrated with another selective noradrenergic reuptake inhibitor, reboxetine [33]. The differential dietary activation of nisoxetine in the arcuate nucleus, however, is of considerable importance because this region is critically involved in the central and peripheral integration of the expression of feeding behavior and body weight controls [34]. Although the exact role of NE in this region had not been delineated, it does appear NE plays a role in regulating neuronal activity of arcuate neurons. Using an in vitro slice preparation, NE application altered the spontaneous discharge rate of arcuate neurons. Most of the responses to NE were excitatory with 61.5% of these neurons increasing their firing rate, 22.9% decreasing their firing rate, and 15.6% were unresponsive. In addition, excitation of the arcuate neurons were mediated by both *α*1-adrenergic and *β*-adrenergic receptors [35]. Hindbrain noradrenergic input also appears to modulate orexigenic arcuate neuronal population. Using saporin-conjugated antidopamine hydroxylase (DSAP), a selective immunotoxin that destroys NE and epinephrine (E) containing neurons, loss of NE/E input in the arcuate nucleus resulted in an increase in baseline levels of neuropeptide Y/agouti-related peptide (NPY/AgRP) mRNA expression. Norepinephrine/epinephrine in the arcuate nucleus appears to regulate glucoprivic feeding response, since animals with DSAP immunotoxin lesions do not increase their food intake in response to 2-deoxy-d-glucose [36]. Studies from Levin and colleagues demonstrated the NE turn-over rate in the arcuate nucleus/median eminence is increased in animals as a result of diet-induced obesity or in rats prone to diet-induced obesity [37, 38]. In this sense, an increase to the feeding suppression to low-dose nisoxetine could have been a result of increased arcuate NE or increased NET availability or both in this region. In addition, one aspect lacking in our study is that we did not determine the phenotype of the c-Fos positive cells. Future work is certainly needed to determine the interaction between diet-induced alterations in arcuate NE, NET availability, and the feeding suppressive and cardiovascular effects of nisoxetine.

## 5. Conclusion

Obesity is associated with neural and metabolic alterations, which are involved in sustaining feeding behavior and increasing body weight. Even though noradrenergic mechanisms involved in feeding and cardiovascular controls are altered with obesity, it remains unclear how the feeding alterations, brain norepinephrine, and cardiovascular alterations interact in the development of obesity. This study used a selective norepinephrine reuptake inhibitor, nisoxetine, to focus on the diet-induced alterations in feeding that emerge with onset of weight gain. Data from these experiments demonstrate that the development of obesity resulted in an increased sensitivity to the feeding suppression effects of nisoxetine and increased nisoxetine-induced neural activation in feeding-related pathways. Our findings have direct implications for treatment strategies of obesity and related metabolic alterations. One thing to note is that the enhanced feeding suppressive effects of low-dose nisoxetine was demonstrated when tested on standard chow. Changes in diet are often accompanied (and are recommended by health care professionals) with the prescribing pharmacotherapy for the treatment of obesity [39]. Therefore, our findings suggest that medications, which act on noradrenergic pathways, may facilitate the feeding suppression needed to reduce food intake and manage obesity, at least in the short-term. Future studies will be conducted to determine the long-term impact of central noradrenergic modulation, food intake, weight management, and cardiovascular control.

## References

[1] Ginsberg HN, Maccallum PR (2009). The obesity, metabolic syndrome, and type 2 diabetes mellitus pandemic: part I. Increased cardiovascular disease risk and the importance of atherogenic dyslipidemia in persons with the metabolic syndrome and type 2 diabetes mellitus. *Journal of the CardioMetabolic Syndrome*.

[2] Szczepanska-Sadowska E, Cudnoch-Jedrzejewska A, Ufnal M, Zera T (2010). Brain and cardiovascular diseases: common neurogenic background of cardiovascular, metabolic and inflammatory diseases. *Journal of Physiology and Pharmacology*.

[3] Ahlskog JE, Hoebel BG (1973). Overeating and obesity from damage to a noradrenergic system in the brain. *Science*.

[4] Kasparov S, Teschemacher AG (2008). Altered central catecholaminergic transmission and cardiovascular disease. *Experimental Physiology*.

[5] Rinaman L (2011). Hindbrain noradrenergic A2 neurons: diverse roles in autonomic, endocrine, cognitive, and behavioral functions. *American Journal of Physiology*.

[6] Ritter S, Pelzer NL, Ritter RC (1978). Absence of glucoprivic feeding after stress suggests impairment of noradrenergic neuron function. *Brain Research*.

[7] Valentino RJ, Curtis AL, Page ME, Pavcovich LA, Florin-Lechner SM (1998). Activation of the locus ceruleus brain noradrenergic system during stress: circuitry, consequences, and regulation. *Advances in Pharmacology*.

[8] Bello NT, Zahner MR (2009). Tesofensine, a monoamine reuptake inhibitor for the treatment of obesity. *Current Opinion in Investigational Drugs*.

[9] Heal DJ, Gosden J, Smith SL (2012). What is the prognosis for new centrally-acting anti-obesity drugs?. *Neuropharmacology*.

[10] Rothman RB, Hendricks EJ (2009). Phentermine cardiovascular safety. *American Journal of Emergency Medicine*.

[11] Rucker D, Padwal R, Li SK, Curioni C, Lau DCW (2007). Long term pharmacotherapy for obesity and overweight: updated meta-analysis. *British Medical Journal*.

[12] Lemberger L, Terman S, Rowe H, Billings R (1976). The effect of nisoxetine (Lilly compound 94939), a potential antidepressant, on biogenic amine uptake in man. *British Journal of Clinical Pharmacology*.

[13] Wong DT, Bymaster FP (1976). The comparison of fluoxetine and nisoxetine with tricyclic antidepressants in blocking the neurotoxicity of p chloroamphetamine and 6 hydroxydopamine in the rat brain. *Research Communications in Chemical Pathology and Pharmacology*.

[14] Cheetham SC, Viggers JA, Butler SA, Prow MR, Heal DJ (1996). [3H]nisoxetine—a radioligand for noradrenaline reuptake sites: correlation with inhibition of [3H]noradrenaline uptake and effect of DSP-4 lesioning and antidepressant treatments. *Neuropharmacology*.

[15] Kelwala S, Stanley M, Gershon S (1983). History of antidepressants: successes and failures. *Journal of Clinical Psychiatry*.

[16] Belej T, Manji D, Sioutis S, Barros HMT, Nobrega JN (1996). Changes in serotonin and norepinephrine uptake sites after chronic cocaine: pre- vs. post-withdrawal effects. *Brain Research*.

[17] Tella SR (1995). Effects of monoamine reuptake inhibitors on cocaine self-administration in rats. *Pharmacology Biochemistry and Behavior*.

[18] Billes SK, Cowley MA (2007). Inhibition of dopamine and norepinephrine reuptake produces additive effects on energy balance in lean and obese mice. *Neuropsychopharmacology*.

[19] Jackson HC, Needham AM, Hutchins LJ, Mazurkiewicz SE, Heal DJ (1997). Comparison of the effects of sibutramine and other monoamine reuptake inhibitors on food intake in the rat. *British Journal of Pharmacology*.

[20] Billes SK, Cowley MA (2008). Catecholamine reuptake inhibition causes weight loss by increasing locomotor activity and thermogenesis. *Neuropsychopharmacology*.

[21] Thomas GH, Babbs AJ, Chatfield RE (2009). 5-HT1A activation counteracts cardiovascular but not hypophagic effects of sibutramine in rats. *Obesity*.

[22] Abolfathi Z, Couture J, Vallée F, LeBel M, Tanguay M, Masson E (2004). A pilot study to evaluate the pharmacokinetics of sibutramine in healthy subjects under fasting and fed conditions. *Journal of Pharmacy and Pharmaceutical Sciences*.

[23] Noh K, Bae K, Min B (2010). Enantioselective pharmacokinetics of sibutramine in rat. *Archives of Pharmacal Research*.

[24] Levin BE (1993). Sympathetic activity, age, sucrose preference, and diet-induced obesity. *Obesity Research*.

[25] Levin BE (1996). Reduced paraventricular nucleus norepinephrine responsiveness in obesity-prone rats. *American Journal of Physiology*.

[26] Levin BE, Dunn-Meynell AA (2002). Maternal obesity alters adiposity and monoamine function in genetically predisposed offspring. *American Journal of Physiology*.

[27] Bello NT, Guarda AS, Terrillion CE, Redgrave GW, Coughlin JW, Moran TH (2009). Repeated binge access to a palatable food alters feeding behavior, hormone profile, and hindbrain c-Fos responses to a test meal in adult male rats. *American Journal of Physiology*.

[28] Paxinos G, Watson C (1998). *The Rat Brain in Stereotaxic Coordinatesed*.

[29] Overton JM, Williams TD, Chambers JB, Rashotte ME (2001). Cardiovascular and metabolic responses to fasting and thermoneutrality are conserved in obese Zucker rats. *American Journal of Physiology*.

[30] Woolard J, Bennett T, Dunn WR, Heal DJ, Aspley S, Gardiner SM (2004). Acute cardiovascular effects of sibutramine in conscious rats. *Journal of Pharmacology and Experimental Therapeutics*.

[31] Birkenfeld AL, Schroeder C, Boschmann M (2002). Paradoxical effect of sibutramine on autonomic cardiovascular regulation. *Circulation*.

[32] Schroeder C, Tank J, Boschmann M (2002). Selective norepinephrine reuptake inhibition as a human model of orthostatic intolerance. *Circulation*.

[33] Miyata S, Hamamura T, Lee Y (2005). Contrasting Fos expression induced by acute reboxetine and fluoxetine in the rat forebrain: neuroanatomical substrates for the antidepressant effect. *Psychopharmacology*.

[34] Schwartz MW, Woods SC, Porte D, Seeley RJ, Baskin DG (2000). Central nervous system control of food intake. *Nature*.

[35] Kang YM, Ouyang W, Chen JY, Qiao JT, Dafny N (2000). Norepinephrine modulates single hypothalamic arcuate neurons via *α*1and *β* adrenergic receptors. *Brain Research*.

[36] Fraley GS, Ritter S (2003). Immunolesion of norepinephrine and epinephrine afferents to medial hypothalamus alters basal and 2-deoxy-D-glucose-induced neuropeptide Y and agouti gene-related protein messenger ribonucleic acid expression in the arcuate nucleus. *Endocrinology*.

[37] Levin BE (1995). Reduced norepinephrine turnover in organs and brains of obesity-prone rats. *American Journal of Physiology*.

[38] Levin BE, Triscari J, Sullivan AC (1986). The effect of diet and chronic obesity on brain catecholamine turnover in the rat. *Pharmacology Biochemistry and Behavior*.

[39] FDA (2001). Guidance for the clinical evaluation of weight-control drugs. *Critical Reviews in Food Science and Nutrition*.

